# Low-molecular-weight chitosan scavenges methylglyoxal and *N*^ε^-(carboxyethyl)lysine, the major factors contributing to the pathogenesis of nephropathy

**DOI:** 10.1186/s40064-015-1106-4

**Published:** 2015-07-03

**Authors:** Chu-Kuang Chou, Shih-Ming Chen, Yi-Chieh Li, Tzu-Chuan Huang, Jen-Ai Lee

**Affiliations:** Chia-Yi Christian Hospital, No. 539 Jhongsiao Rd., Chia-Yi City, 60002 Taiwan; School of Pharmacy, College of Pharmacy, Taipei Medical University, No. 250 Wuxing St., Taipei, 11031 Taiwan, ROC; Department of Internal Medicine, National Taiwan University Hospital, No. 7 Chung-Shan South Road, Taipei City, 10002 Taiwan

**Keywords:** Nephropathy, Methylglyoxal, *N*^ε^-(carboxyethyl)lysine, Low-molecular-weight chitosan

## Abstract

Methylglyoxal (MG) can cause protein glycation, resulting in cell damage and dysfunction. Accumulation of MG and its downstream metabolite *N*^ε^-(carboxyethyl)lysine (CEL) has been identified in several variations of nephropathy, including diabetic, hypertensive, and gentamicin-induced nephropathies. In this study, we investigated the effects of low-molecular-weight chitosan (lmw-chitosan) on MG-induced carbonyl stress in aristolochic acid-induced nephropathy. We used a buffer to investigate whether MG could be scavenged by lmw-chitosan in vitro. In addition, we also used a mouse model of aristolochic acid-induced nephropathy, which exhibits 12-fold greater accumulation of MG in the kidneys than that found in control animals, to examine whether lmw-chitosan could decrease MG levels in vivo. Examination of the binding of lmw-chitosan with MG in vitro demonstrated that the concentration of lmw-chitosan necessary to achieve 50% inhibition was 4.60 µg mL^−1^. Treatment with lmw-chitosan (500 mg kg^−1^ day^−1^ orally) for 14 days significantly decreased renal MG accumulation from 212.86 ± 24.34 to 86.15 ± 33.79 µg g^−1^ protein (*p* < 0.05) and CEL levels from 4.60 ± 0.27 to 2.84 ± 0.28 µmol µg^−1^ protein (*p* < 0.05) in the aristolochic acid-induced nephropathy model. These data suggest that lmw-chitosan might represent a novel treatment modality for MG-related diseases such as nephropathy.

**Electronic supplementary material:**

The online version of this article (doi:10.1186/s40064-015-1106-4) contains supplementary material, which is available to authorized users.

## Background

Nephropathy can lead to the acquisition of end-stage renal disease, which is associated with major health problems. Causes of nephropathy include medications or disease, as well as the toxicity associated with compounds such as the organic by-product methylglyoxal (MG). Aristolochic acid (AAN)- and gentamicin-induced nephropathies are both related to MG accumulation in the kidney (Li et al. 2012, 2013). The pathogenesis of diabetic nephropathy and its associated complications are primarily related to MG and its downstream metabolites, which cause injury to the kidneys (Rabbani and Thornalley 2011). Additionally, increased levels of MG and advanced glycation end products (AGEs) have been identified in the kidneys of spontaneously hypertensive rats (Wang et al. 2004). MG can also cause oxidative stress. MG is generated during the process of tissue injury repair, which places additional energy demands on cells. MG, formed as a byproduct of glycolysis and lipid and amino acid metabolism, modifies proteins and interferes with protein function (Rabbani and Thornalley 2012). Furthermore, *N*^ε^-(carboxyethyl)lysine (CEL), the downstream AGE of MG, is also known to enhance inflammation (Rabbani and Thornalley 2011). To date, only metformin has been approved as an effective MG-reducing agent by the US Food and Drug Administration. However, this drug is not suggested for use in patients with renal insufficiency due to its toxicity. Therefore, an effective and potent anti-MG agent is needed for better prevention and treatment of MG-induced renal injury.

Chitosan is a natural polysaccharide with a high molecular weight (500–1,000 kDa) and has been widely used in the pharmaceutical, food, and cosmetic industries (Zhang et al. 2010). Upon hydrolyzation into low-molecular-weight chitosan (lmw-chitosan), it becomes highly water-soluble and therefore has more biological applications. For example, lmw-chitosan can be utilized as an antibacterial agent, antifungal agent, antidiabetic drug, and as a lipid-lowering medication (Lee et al. 2003; Seyfarth et al. 2008; Anraku et al. 2010; Chen et al. 2012). In addition, lmw-chitosan has been shown to have protective effects on the kidneys in studies on diabetes (Yoon et al. 2008). However, the molecular mechanisms underlying these effects have not been elucidated.

Lmw-chitosan can also be used as an agent for kidney-specific targeting and drug delivery. For example, conjugation of lmw-chitosan nanoparticles with drugs that benefit the kidney such as catechol or tripterygium glycoside have been investigated by multiple laboratories (Qiao et al. 2014; Chen et al. 2013). Conversely, the demonstration that the levels of MG are increased 12-fold in the kidneys of AAN mice suggests that this might represent a good model for investigating the effect of lmw-chitosan on decreasing physiological MG accumulation in vivo.

While MG and CEL are important in disease pathogenesis, the relationships between lmw-chitosan and MG or CEL have not been determined. Therefore, in this study we aimed to elucidate the effects and underlying mechanisms of lmw-chitosan on MG and CEL accumulation in vitro and in vivo.

## Results

### Inhibitory activity of lmw-chitosan in vitro and in vivo

**Figure 1:**
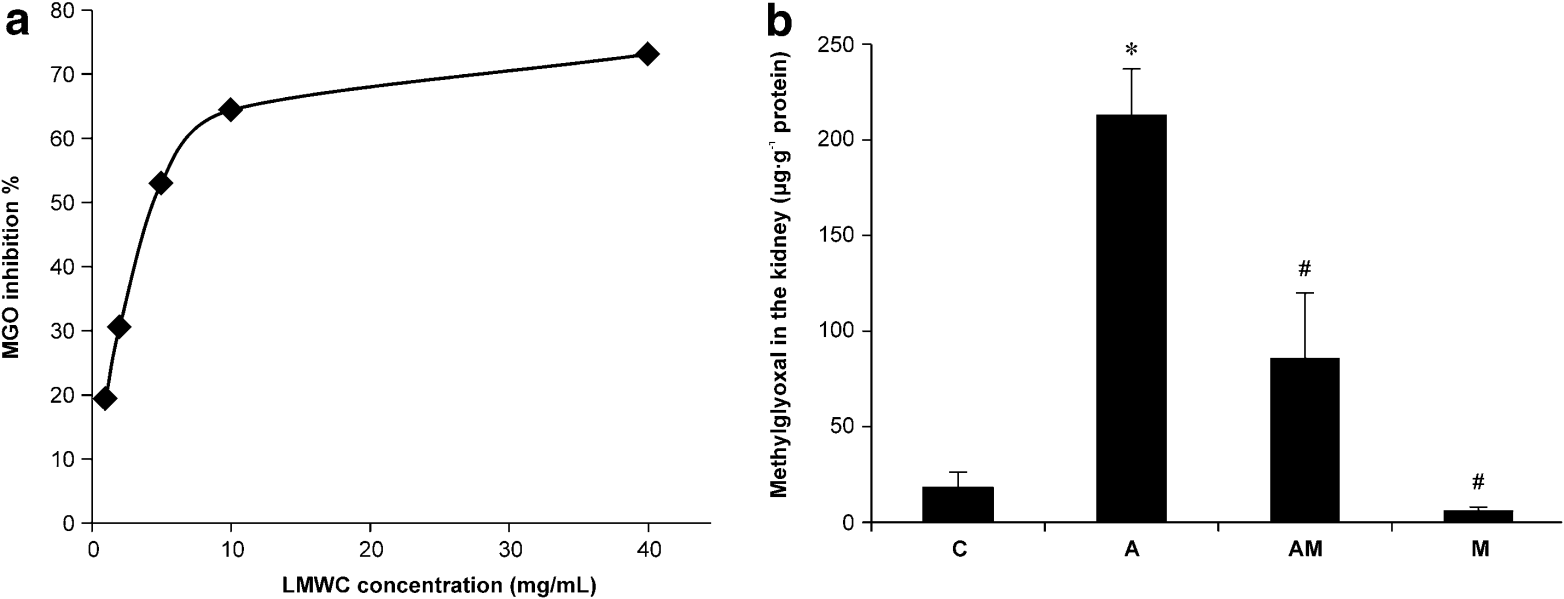
Inhibitory effects of lmw-chitosan on MG in vitro and in vivo. **a** Lmw-chitosan binds to MG and decreases the level of MG in vitro. The inhibition curve of lmw-chitosan on MG binding is shown. The concentration necessary to achieve 50% inhibition was 4.60 µg mL^−1^, n = 3. **b** *Group C* represents the control group. *Group A* was induced by AA (10 mg kg^−1^ day^−1^) for 5 days. *Group AM* represents lmw-chitosan-treated AAN mice. *Group M* represents normal mice treated with lmw-chitosan. MG levels were observed to increase in AAN mice; however, this increase is reversed in lmw-chitosan-treated AAN mice. *p < 0.05, compared to *Group C*; ^#^ *p* < 0.05, compared to *Group A*, n = 5–6.

The data of MG chelation and inhibition by lmw-chitosan in vitro are shown in Figure 1a. The concentration of lmw-chitosan required for 50% inhibition was determined to be 4.60 µg mL^−1^. MG levels in the in vivo study were ascertained by HPLC and then normalized with protein assay. MG levels in the kidneys of Group A mice were significantly higher than those in Group C mice (212.86 ± 24.34 vs. 18.23 ± 8.05 μg g^−1^ protein, respectively, *p* < 0.05). Treatment with lmw-chitosan decreased MG levels to 86.15 ± 33.79 μg g^−1^ protein (*p* < 0.05 vs. Group AM; Figure 1b). Additionally, the lmw-chitosan only group (Group M) exhibited an MG level of 6.26 ± 1.79 μg g^−1^ protein.

### Immunohistological staining and quantitative detection of CEL

**Figure 2:**
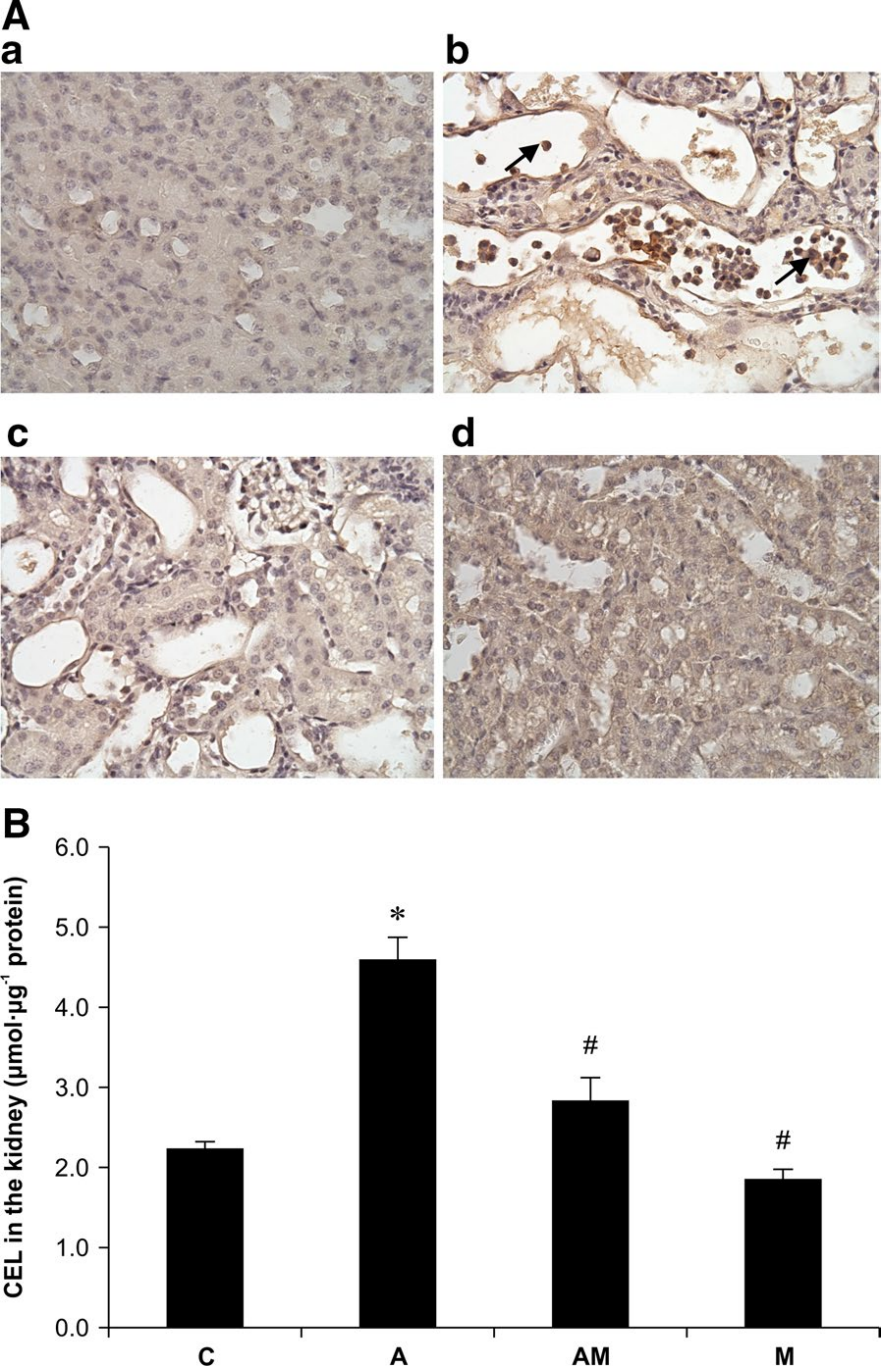
Effects of lmw-chitosan on CEL concentrations in renal tissues of mice. **A** Immunostaining of CEL in the mouse kidney. **a** Control group (*Group C*); **b** mice induced by AA (10 mg kg^−1^ day^−1^) for 5 days (*Group A*); **c** lmw-chitosan-treated AAN mice (*Group AM*); and **d** normal mice treated with lmw-chitosan (*Group M*). CEL staining can be observed in the tubular region of monocytes (*arrow*). Nuclei were counterstained with hematoxylin. Original magnification: ×400 . **B** ELISA of CEL levels in mouse kidneys. *Group C* represents the control group. *Group A* was induced by AA (10 mg kg^−1^ day^−1^) for 5 days. *Group AM* represents lmw-chitosan-treated AAN mice. *Group M* consisted of normal mice treated with lmw-chitosan. It can be observed that CEL was increased by threefold in *Group A*, and that this effect was reversed in *Group AM*. *p < 0.05, compared to *Group C*; ^#^ *p* < 0.05, compared to the *Group A*, n = 5–6.

Immunochemistry was performed to visualize CEL expression in the kidneys (Figure 2A). We observed that CEL staining was present in several monocytes of the desquamation tubule region and that lmw-chitosan treatment significantly decreased the level of CEL staining (*p* < 0.05).

The quantitative data of CEL expression in mouse kidneys are shown in Figure 2B. The levels of CEL in Group A mice were significantly higher than those in Group C mice (4.6 ± 0.27 vs. 2.24 ± 0.08 μmol µg^−1^ protein, respectively). After lmw-chitosan treatment, the CEL levels in Group AM mice decreased to 2.84 ± 0.28 μmol/µg protein (*p* < 0.05 vs. Group A). Additionally, the lmw-chitosan only group (Group M) had a CEL level of 1.86 ± 0.12 μmol µg^−1^ protein.

### Effects of lmw-chitosan on glutathione levels in the kidneys of AAN mice

**Figure 3:**
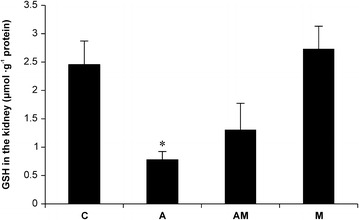
Effects of lmw-chitosan on glutathione concentrations in mouse kidney. *Group C* represents the control group. *Group A* was induced by AA (10 mg kg^−1^ day^−1^) for 5 days. *Group AM* represents lmw-chitosan-treated AAN mice. *Group M* consists of normal mice treated with lmw-chitosan. Glutathione levels decreased in the kidneys of AAN mice; treatment with lmw-chitosan did not significantly reverse this effect. *p < 0.05, compared to *Group C*; ^#^ *p* < 0.05, compared to the Group A, n = 5–6.

Glutathione is an important anti-oxidant, and the relative level of glutathione in a cell, compared to the level of its oxidized form, serves as an indicator of oxidative stress. In addition, glutathione is a required cofactor during MG detoxification. We determined that the intrarenal glutathione levels in Group A mice were lower than those in Group C mice (0.78 ± 0.15 vs. 2.46 ± 0.41 μg g^−1^ protein, respectively; *p* < 0.05; Figure 3); however, glutathione levels did not recover significantly after administration of lmw-chitosan treatment (Group AM: 1.31 ± 0.46 μg g^−1^ protein, *p* > 0.05 vs. Group A). Additionally, the lmw-chitosan only group (Group M) demonstrated a glutathione level of 2.73 ± 0.40 μg g^−1^ protein.

### Hypothetical scheme of the role of lmw-chitosan in disease prevention

**Figure 4:**
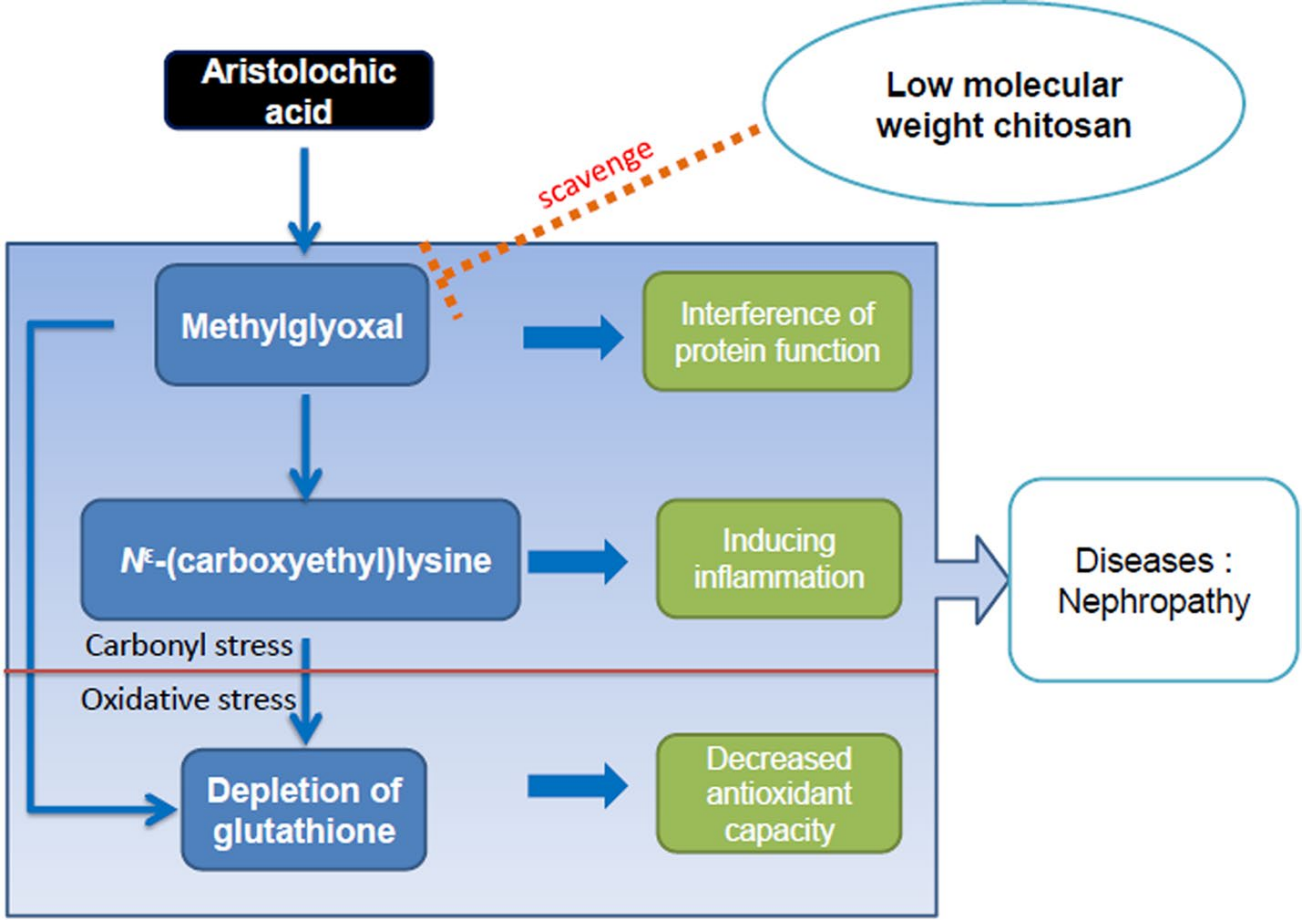
The protective mechanism of lmw-chitosan. Lmw-chitosan can block MG and CEL accumulation and might improve the damage resulting from protein dysfunction and inflammation. Interactions with carbonyl and oxidative stressors could promote an increase in overall stress. However, the role of lmw-chitosan on carbonyl stress is more vital than that on oxidative stress.

Figure 4 illustrates a mechanism by which MG accumulation leads to disease and serves as a target for lmw-chitosan binding. Initially, elevated levels of MG and CEL cause significant carbonyl stress in AA-injected mice and in diseases such as nephropathy, and subsequent administration of lmw-chitosan can successfully reverse the increase in MG and CEL levels. However, the lack of response in our animal model suggests that the glutathione level does not represent the primary target of lmw-chitosan; instead, we suggest that the reversal of MG and MG-derived CEL build-up by lmw-chitosan is the key mechanism of this treatment against nephropathy.

## Discussion

In this study, we investigated the effects of lmw-chitosan on carbonyl stress in mice with AAN. We found that AAN mice treated with lmw-chitosan showed improved renal function, and that lmw-chitosan treatment significantly decreased renal MG accumulation and CEL levels. Thus, our data demonstrated that lmw-chitosan could decrease renal damage in AAN by ameliorating the accumulation of MG and CEL in the kidney.

Furthermore, in a previous study, a clinical trial was conducted to characterize the effects of chitosan administration in patients with renal failure that were undergoing long-term stable hemodialysis treatment. The data revealed that patients receiving chitosan (4,050 mg day^−1^) for 4 weeks had significantly lower blood urea nitrogen and serum creatinine levels than those in patients not receiving chitosan (Jing et al. 1997). Similarly, we found that lmw-chitosan ameliorated clinical chemistry parameters and improved histology in our mouse model (Additional files 1, 2: Figures S1, S2).

MG can glycate proteins at a significantly higher level than can glucose (Thornalley 2005); in addition, MG-mediated protein modification can alter the biological activity of the glycated protein and produce toxic AGEs. Oxidative stress induced by MG can also cause cell damage. Thus, MG has a higher impact than blood glucose on diabetic neuralgia, and regulating MG levels is an important preventative measure against the development of diabetic complications (Bierhaus et al. 2012).

Several pharmacological interventions for preventing MG-related injuries have been proposed, and several chemical agents, including thiamine (Rabbani et al. 2009) and aminoguanidine (Thornalley 2003), have been tested. However, most of these agents cannot be used for treatment because of their toxicity, instability, or low potency. Among all the available agents, only metformin is widely used for the treatment of diabetes and for lowering blood sugar. However, metformin is not suggested for use in patients with renal function insufficiency because of the risk of lactic acidosis, a life-threatening side effect of metformin that occurs only in 8 out of every 1,00,000 patients but has a mortality rate of up to 50%. Despite this drawback of metformin, because no effective MG inhibitors are currently available, doctors still use metformin to treat MG-related nephropathy. Therefore, the development of a novel, safe, and effective MG inhibitor is urgently needed. In contrast, lmw-chitosan has been used widely in the pharmaceutical industry and was introduced in humans as a lipid-lowering agent (Choi et al. 2012); there have been no adverse effects of chitosan reported to date. Therefore, we propose that lmw-chitosan might be an ideal supplementary strategy for preventing MG-related complications in humans.

In addition to its impact on diabetes, MG was recently found to be related to the pathogenesis of numerous other diseases. Ahmed et al. (2014) showed that MG and MG-derived AGEs might be increased in arthritis. Additionally, Tikellis et al. (2014) increased the plasma MG levels of mice to the levels observed in diabetic mice by addition of 1% MG to the drinking water and by inhibiting its primary clearance enzyme, glyoxalase I. Euglycemic mice with increased MG levels showed increased vascular adhesion, inflammation, and augmented atherogenesis, and the pathophysiology was similar to that observed in hyperglycemic mice with diabetes. Therefore, it was suggested that MG plays an important role in the development of inflammation- and physiology-related diseases.

In our present study, we show that MG can be chelated by lmw-chitosan in vitro, and demonstrate that lmw-chitosan can also lower the MG levels in renal tissue in vivo in an animal model, which might prevent renal injury by AA. Limitations of our study include that the question of whether MG is enzymatically metabolized by lmw-chitosan or binds to lmw-chitosan remains unanswered, and that the effects and toxicities of the end products from MG and lmw-chitosan association are also not clear. Further study is needed to address these issues.

## Conclusions

In conclusion, our finding that lmw-chitosan could decrease the accumulation of MG and CEL might be valuable for understanding the mechanisms underlying the bioactivities of chitosan. Furthermore, our data demonstrated that lmw-chitosan might be useful for the treatment of severe renal diseases, although further evidence is needed to confirm this.

## Methods

### Chemicals

Crab shell chitosan (84% deacetylated; approximately 1,100 kDa) was obtained from Ohka Enterprises Co. Ltd. (Kaohsiung, Taiwan). A CEL detection kit was obtained from Cell Biolabs Inc. (San Diego, CA, USA). A glutathione assay kit was obtained from Cayman Chemical Co. (Ann Arbor, MI, USA). Anti-CEL antibodies were purchased from Cosmo Bio Co. (Tokyo, Japan). A polymer detection system, which includes blocking reagent A and B, was purchased from Nichirei Biosciences Inc. (Tokyo, Japan).

### Preparation of chitosanase and lmw-chitosan

Chitosan was used to induce bamboo chitosanase, which was in turn utilized to digest chitosan (1,100 kDa) into lmw-chitosan (~29 kDa), as previously described (Chang et al. 2011). Briefly, bamboo (*Bambusa oldhamii*) shoots were coated with chitosan and stored at 25°C to induce chitosanase, which was isolated, and its activity was measured. Chitosan was suspended in 4.5% acetic acid, digested with 180 U chitosanase at 50°C for 18 h, and purified before use.

### Determination of the inhibitory effects of lmw-chitosan on MG in vitro and in kidney samples

MG levels were determined using HPLC. Lmw-chitosan was diluted to different concentrations (1, 2, 5, 10, and 40 µg mL^−1^) for testing the MG inhibition effect in vitro. In addition, kidney homogenates were diluted fourfold, and 20 μL diluted homogenate was used to detect the MG level. To each sample, 2 μL ammonium chloride buffer (pH 10) was added to create an alkaline environment for the derivatization reaction. The derivative reagent, 50 µL 7.5 × 10^−4^ M DDP, was added to each sample, and the reaction mixture was incubated at 60°C for 30 min in the dark. The reaction was then stopped by addition of 500 µL 0.01 M citric acid buffer (pH 6). The mixture was vortexed, spun down, incubated on ice for 10 min, and then centrifuged at 12,000 rpm for 10 min at 4°C. The supernatant was filtered with a 0.45 µm filter to remove impurities and then layered onto an ODS column for separation at 33°C (Biosil, 250 mm × 4.6 mm ID; 5 μm particle size; Biosil Chemical Co. Ltd., Taipei, Taiwan). Acetonitrile was used as the mobile phase: 0.01 M citric acid buffer = 3:97, v/v; flow rate = 0.7 mL/min, Ex/Em = 330/500 nm. All samples were analyzed within 24 h.

### Animal treatment and sample preparation

A total of 23 six-week-old female C3H/He mice were purchased from the National Laboratory Animal Breeding and Research Center (Taipei, Taiwan). The mice were divided into three groups of six mice each plus a control group consisting of five animals after 1 week of acclimation. The control (Group C) and lmw-chitosan only (Group M) groups were injected with 0.1 mL normal saline each day for 5 days (days 1–5, IP), whereas the disease (Group A) and therapy (Group AM) groups were injected with 0.1 mL 10 mg kg^−1^ AA (IP) each day for 5 days. The mice in Groups AM and M then received lmw-chitosan (500 mg kg^−1^ day^−1^, PO) for 14 days after AA injection, while mice in Groups C and A received the same volume of water. The mice were anesthetized with isoflurane and sacrificed by drawing blood from the inferior vena cava. The kidneys were then harvested and immediately put on ice. The kidneys were homogenized with a motorized homogenizer unit at 6,000 rpm by using phosphate buffered saline (PBS); the resulting homogenates were stored at −80°C until use.

### Determination of CEL concentrations in kidney samples

The CEL concentrations were determined using a commercial kit. Briefly, all kidney homogenate samples were diluted in PBS to a final total protein content of 10 μg mL^−1^. One hundred microliters of each sample or standard was then added to a protein adsorbent plate, which was incubated overnight at 4°C. After incubation, the plate was washed with PBS twice and subsequently incubated with 200 µL Assay Diluent buffer for 1.5 h. The plate was then washed three times with 250 μL wash buffer and incubated with anti-CEL antibodies at room temperature for 1 h on an orbital shaker. Then the plate was again washed with wash buffer three times and incubated with secondary horseradish peroxidase-conjugated antibodies for 1.5 h at room temperature on an orbital shaker. Next, the plate was washed with wash buffer five times and then incubated with 100 μL Substrate Solution at room temperature for 15 min on an orbital shaker. The enzyme reaction was stopped by adding 100 μL Stop Solution to each well. The absorbances of the reaction mixtures were read immediately on a microplate reader at 450 nm. Reduced bovine serum albumin was used as an assay blank.

### Immunochemical staining of CEL

To determine the localization of CEL by immunohistochemistry, mouse kidneys were fixed with 4% paraformaldehyde for 2 days, dehydrated with different concentrations of ethanol and xylene, embedded in paraffin, and sectioned to 5 μm thickness. After blocking endogenous peroxidase activity by treating the sections with 3% H_2_O_2_ for 10 min, slides were washed three times with PBS. The slides were then incubated with blocking reagent A for 60 min at room temperature. Anti-CEL antibodies were diluted 50-fold and incubated with the samples overnight, followed by washing with PBS. Next, the samples were incubated with blocking reagent B for 10 min at room temperature. The samples were then incubated with a secondary antibody for 10 min at room temperature. Subsequently, the tissue sections were washed for a final three times and then dried carefully. Two drops of the chromogen/substrate reagent was added to each section, and the samples were incubated in the dark at room temperature for 10 min. Sections were then counterstained with hematoxylin. Staining without inclusion of the primary antibody was performed as the negative control.

### Determination of glutathione concentrations in kidney samples

The glutathione levels in the kidney samples were measured using a glutathione assay kit (Cayman), as previously described (Li et al. 2012). Briefly, the tissue homogenates were added to a cocktail buffer containing glutathione reductase, glucose-6-phosphate dehydrogenase, and 5,5′-dithiobis-(2-nitrobenzoic acid) (DTNB). The mixture was incubated at 37°C in the dark, and the absorbance was measured at 405 nm at 5-min intervals over 30 min.

### Statistical analysis

All data were expressed as means ± standard errors of the means (SEMs, where n ≤ 5). The differences were analyzed using one-way analysis of variance (ANOVA), and the levels of significance among various treatments were determined using Scheffe’s multiple range test. Differences with *p* values < 0.05 were considered statistically significant.

## Additional files

Additional file 1:
**Figure S1.** Renal function changes following treatment with lmw-chitosan. Renal function is shown. The white bar represents CCr levels (mL min^−1^ kg^−1^), and the gray bar represents BUN levels (mg dL^−1^) after treatment with lmw-chitosan. Group C represents the control group. Group A was induced by AA (10 mg kg^−1^ day^−1^) for 5 days. Group AM represents lmw-chitosan-treated AAN mice. Group M represents normal mice treated with lmw-chitosan. Additional file 2:
**Figure S2.** Renal histological changes following treatment with lmw-chitosan. Sections from rat kidneys stained with hematoxylin and eosin were imaged by light microscopy. (A) Histology of kidney tissues in the control group. (B) A large number of necrotic tubules and desquamation after treatment with 10 mg kg^−1^ day^−1^ AA for 5 days. (C) Treatment of AAN mice with 500 mg kg^−1^ day^−1^ lmw-chitosan for 14 days resulted in a significant improvement in histology. (D) The administration of 500 mg kg^−1^ day^−1^ lmw-chitosan for 14 days.

## Abbreviations

Lmw-chitosan: low molecular weight-chitosan
AA: aristolochic acid
AAN: aristolochic acid nephropathy
MG: methylglyoxal
AGEs: advanced glycation end products
ANOVA: analysis of variance
CEL: *N*^ε^-(carboxyethyl)lysine
DAB: 3,3′-diaminobenzidine
DDP: diaminedichloroplatinum(II)
DTNB: 5,5′-dithiobis-(2-nitrobenzoic acid)
ELISA: enzyme-linked immunosorbent assay
Ex/Em: Excitation/emission
HPLC: high-performance liquid chromatography
ID: inner diameter
IP: intraperitoneal injection
ODS: octadecylsilyl group
PBS: phosphate buffered saline
PO: per os
SEMs: standard errors of the means
