# Supplementary figures and images for: Low-molecular-weight chitosan scavenges methylglyoxal and Nε-(carboxyethyl)lysine, the major factors contributing to the pathogenesis of nephropathy
Source: Springerplus. 2015 Jul 3;4:312. doi: 10.1186/s40064-015-1106-4 (PMC4489968; doi:10.1186/s40064-015-1106-4)

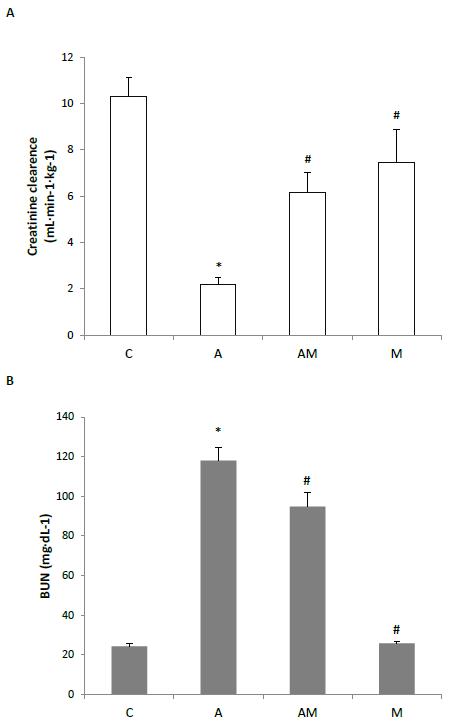

Supplement: Additional file 1: — Figure S1. Renal function changes following treatment with lmw-chitosan. Renal function is shown. The white bar represents CCr levels (mL min−1 kg−1), and the gray bar represents BUN levels (mg dL−1) after treatment with lmw-chitosan. Group C represents the control group. Group A was induced by AA (10 mg kg−1 day−1) for 5 days. Group AM represents lmw-chitosan-treated AAN mice. Group M represents normal mice treated with lmw-chitosan. [file 40064_2015_1106_MOESM1_ESM.tiff]

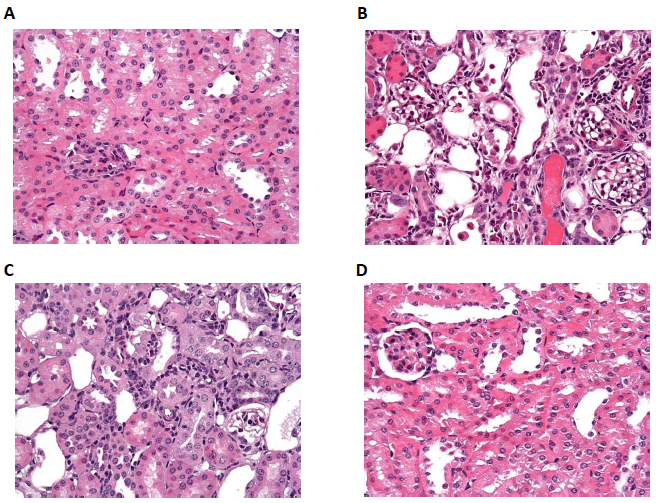

Supplement: Additional file 2: — Figure S2. Renal histological changes following treatment with lmw-chitosan. Sections from rat kidneys stained with hematoxylin and eosin were imaged by light microscopy. (A) Histology of kidney tissues in the control group. (B) A large number of necrotic tubules and desquamation after treatment with 10 mg kg−1 day−1 AA for 5 days. (C) Treatment of AAN mice with 500 mg kg−1 day−1 lmw-chitosan for 14 days resulted in a significant improvement in histology. (D) The administration of 500 mg kg−1 day−1 lmw-chitosan for 14 days. [file 40064_2015_1106_MOESM2_ESM.tiff]
